# Supplementary material for: Spatial Regulation of CAR Signaling Enables Logic-Gated Activity
Source: bioRxiv. 2026 May 24:2026.05.22.726983. Preprint. [Version 1] doi: 10.64898/2026.05.22.726983 (PMC13228326; doi:10.64898/2026.05.22.726983)
Supplement: 1 [file NIHPP2026.05.22.726983v1-supplement-1.pdf]

bioRxiv preprint doi: https://doi.org/10.64898/2026.05.22.726983; this version posted May 24, 2026. The copyright holder for this preprint (which was not certified by peer review) is the author/funder, who has granted bioRxiv a license to display the preprint in perpetuity. It is made available under a CC-BY 4.0 International license.

Supplementary Figures

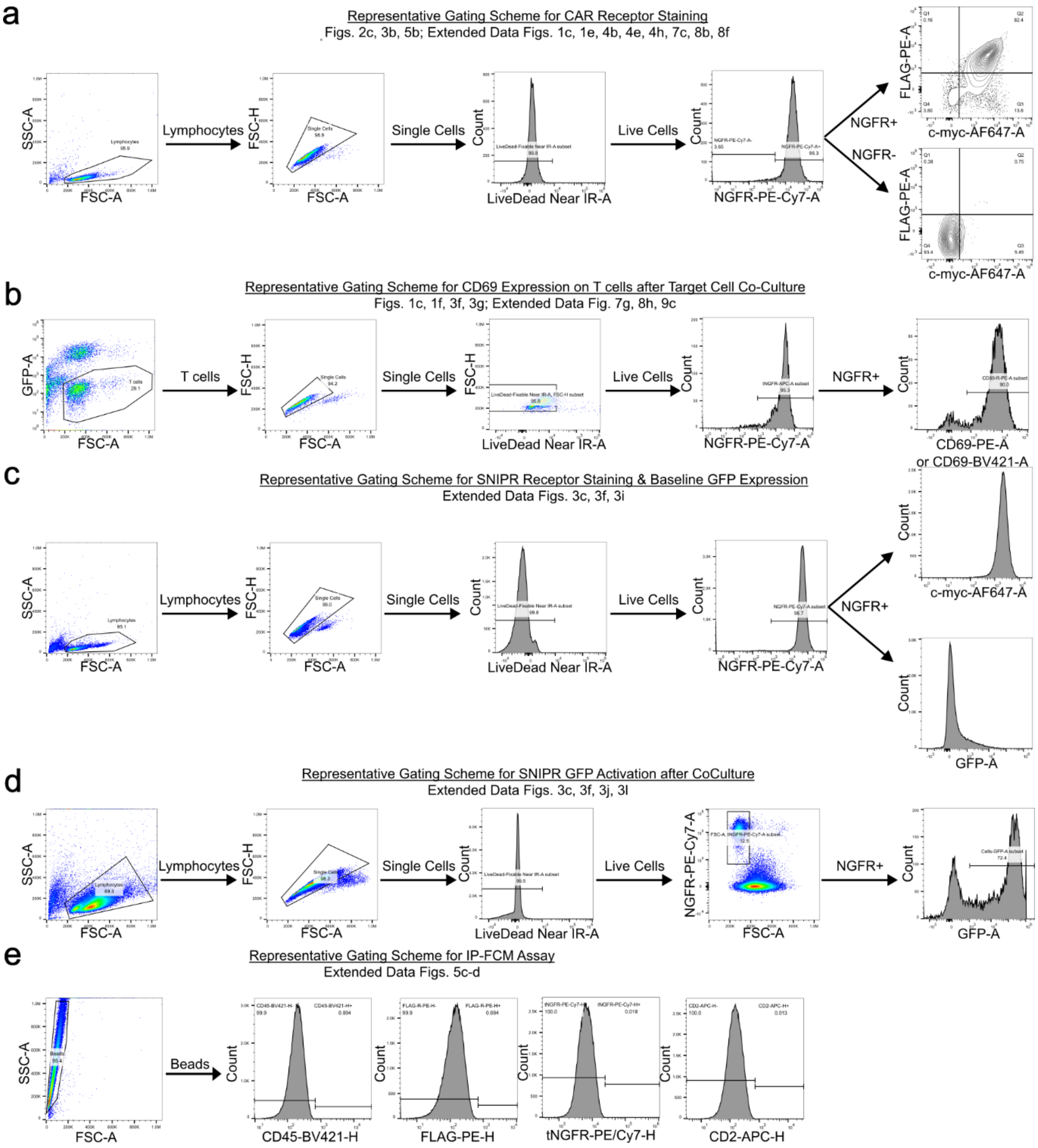

Supplementary Figure S1. **(a-e)** Flow cytometry gating schemes for various *in vitro* experiments.

bioRxiv preprint doi: https://doi.org/10.64898/2026.05.22.726983; this version posted May 24, 2026. The copyright holder for this preprint (which was not certified by peer review) is the author/funder, who has granted bioRxiv a license to display the preprint in perpetuity. It is made available under a CC-BY 4.0 International license.

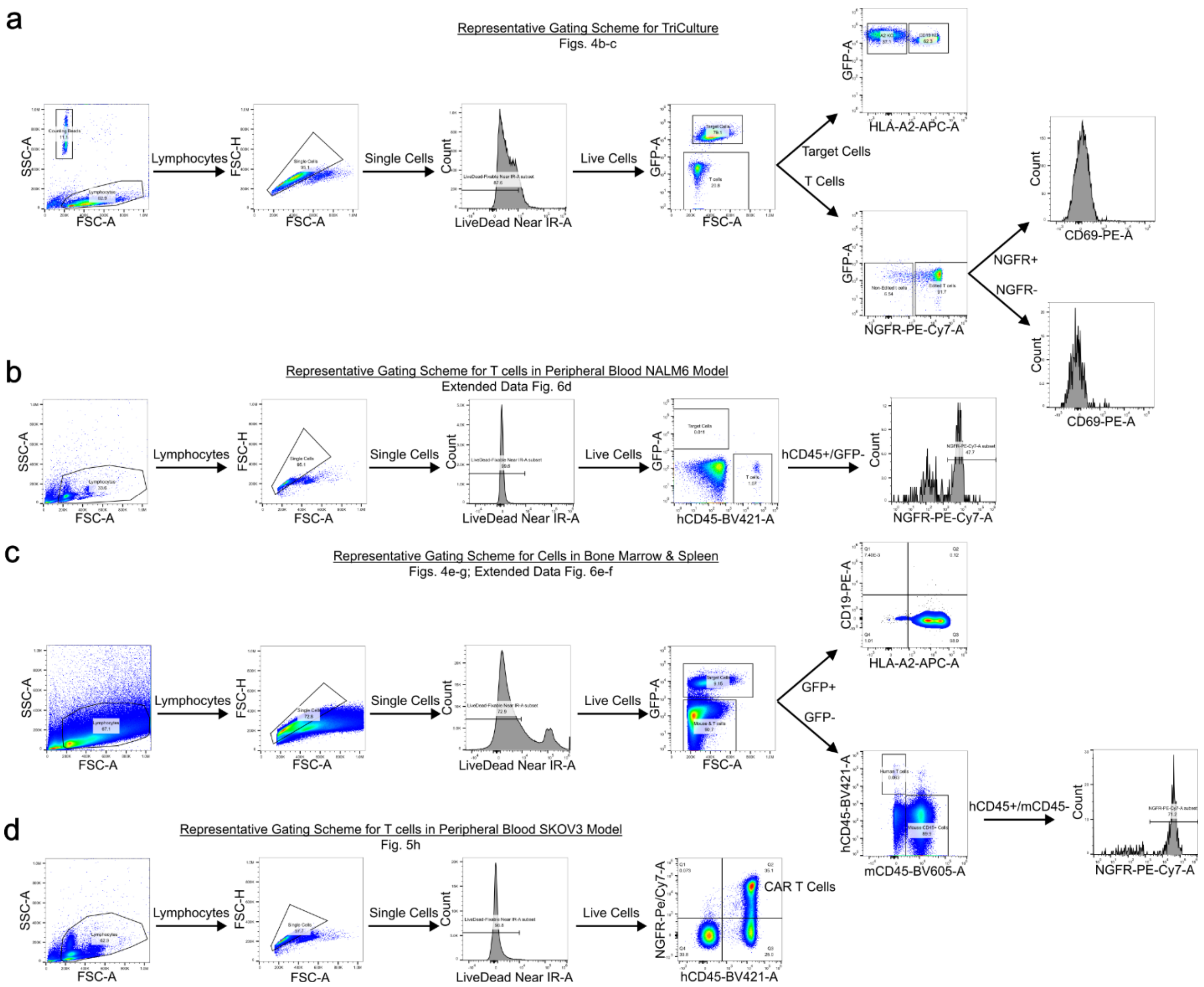

Supplementary Figure S2. **(a-d)** Flow cytometry gating schemes for mixed co-culture and *in vivo* experiments.
